# Nucleus basalis of Meynert degeneration precedes and predicts cognitive impairment in Parkinson’s disease

**DOI:** 10.1093/brain/awy072

**Published:** 2018-03-21

**Authors:** Jonathan Schulz, Gennaro Pagano, Juan Alberto Fernández Bonfante, Heather Wilson, Marios Politis

**Affiliations:** Neurodegeneration Imaging Group, Institute of Psychiatry, Psychology and Neuroscience, King’s College London, London, UK

**Keywords:** nucleus basalis of Meynert, MRI, DTI, cognitive decline, Parkinson’s disease

## Abstract

Currently, no reliable predictors of cognitive impairment in Parkinson’s disease exist. We hypothesized that microstructural changes at grey matter T_1_-weighted MRI and diffusion tensor imaging in the cholinergic system nuclei and associated limbic pathways underlie cognitive impairment in Parkinson’s disease. We performed a cross-sectional comparison between patients with Parkinson’s disease with and without cognitive impairment. We also performed a longitudinal 36-month follow-up study of cognitively intact Parkinson’s disease patients, comparing patients who remained cognitively intact to those who developed cognitive impairment. Patients with Parkinson’s disease with cognitive impairment showed lower grey matter volume and increased mean diffusivity in the nucleus basalis of Meynert, compared to patients with Parkinson’s disease without cognitive impairment. These results were confirmed both with region of interest and voxel-based analyses, and after partial volume correction. Lower grey matter volume and increased mean diffusivity in the nucleus basalis of Meynert was predictive for developing cognitive impairment in cognitively intact patients with Parkinson’s disease, independent of other clinical and non-clinical markers of the disease. Structural and microstructural alterations in entorhinal cortex, amygdala, hippocampus, insula, and thalamus were not predictive for developing cognitive impairment in Parkinson’s disease. Our findings provide evidence that degeneration of the nucleus basalis of Meynert precedes and predicts the onset of cognitive impairment, and might be used in a clinical setting as a reliable biomarker to stratify patients at higher risk of cognitive decline.

## Teaser

There are currently no reliable predictors of cognitive impairment in Parkinson’s disease. Using both cross-sectional and longitudinal comparisons, Schulz *et al.* report that grey matter volume loss and increased mean diffusivity in the nucleus basalis of Meynert is predictive of cognitive impairment in Parkinson’s disease.

## Introduction

When James Parkinson first described the ‘shaking palsy’ in 1871, he assumed that ‘the senses and intellect were uninjured’ (Parkinson, 1817). Unfortunately, this claim was not fully accurate (Saeed *et al.*, 2017). Cognitive impairment is highly prevalent in Parkinson’s disease, and approximately 80% of patients with Parkinson’s disease will eventually develop dementia during the course of their illness (Hely *et al.*, 2008). Cognitive impairment is one of the most clinically relevant symptoms in Parkinson’s disease (Aarsland *et al.*, 2012) and causes an increased risk of mortality and significant reduction in quality of life (Forsaa *et al.*, 2010; Winter *et al.*, 2011).

The mechanisms underlying the development of cognitive impairment in Parkinson’s disease remain unclear. Several imaging and clinical markers have been evaluated over the past years as potential predictors for the development of cognitive impairment in Parkinson’s disease (Moore and Barker, 2014; Xu *et al.*, 2016). Clinical markers for predicting cognitive impairment in Parkinson’s disease vary across studies with contradicting evidence. Depression, rapid eye movement sleep behaviour disorder (Aarsland *et al.*, 2003; Zhu *et al.*, 2014), gait dysfunction, cerebrovascular diseases associated with white matter lesions (Ma *et al.*, 2015), olfactory dysfunction, *APOE* genotype, and CSF amyloid-β:tau ratio (Schrag *et al.*, 2017) have been suggested as predictors for the development of cognitive impairment in Parkinson’s disease (Aarsland *et al.*, 2003); however, several of these have been disputed (Zhu *et al.*, 2014). Imaging studies have shown cortical and subcortical brain regions to be predictive of cognitive impairment in Parkinson’s disease, including structural and microstructural changes within the entorhinal cortex, amygdala, hippocampus, insula, thalamus, striatum and tempo-parieto-frontal areas (Hattori *et al.*, 2012; Melzer *et al.*, 2012). However, evidence from these studies are limited due to small sample sizes, caveats in study designs, classification of cognitive impairment, and subject inclusion criteria.

Loss of cholinergic innervation of the cerebral cortex has been suggested as one mechanism of dementia (Aarsland *et al.*, 2017) and pathological changes seen in patients with Parkinson’s disease with cognitive impairment support this theory. In the basal forebrain, extra-nigral Lewy bodies are present in neurons of the nucleus basalis of Meynert, the primary source of cholinergic innervation of the cerebral cortex (Bohnen and Albin, 2011*a*). Braak *et al.* (2003) suggest this cholinergic neuron degeneration occurs at the same stage as nigral pathology. Imaging studies have demonstrated structural and microstructural changes within the nucleus basalis of Meynert to be predictive of cognitive impairment (Lee *et al.*, 2014; Schmitz and Nathan Spreng, 2016). Significant loss of cholinergic neurons in the nucleus basalis of Meynert has been observed in Parkinson’s disease in the absence of Alzheimer’s disease pathology (Rogers *et al.*, 1985), and PET imaging studies have confirmed that cholinergic neurons in the basal forebrain degenerate at early stages of Parkinson’s disease. This degeneration progresses with the onset of cognitive impairment (Bohnen and Albin, 2011*a*). Lower levels of choline acetyltransferase and acetylcholinesterase have also been associated with cognitive impairment in Parkinson’s disease, at similar levels as seen in Alzheimer’s disease (Bohnen *et al.*, 2003). Currently, no robust predictors of cognitive impairment are validated and used in clinical practice.

**Figure 1:**
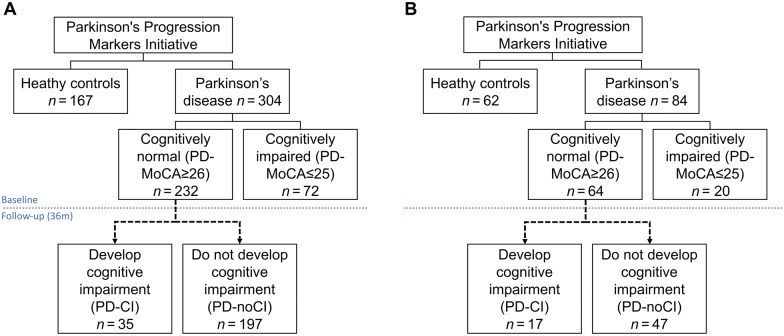
**Study population.** (**A**) Healthy controls and patients with Parkinson’s disease included for grey matter analysis. (**B**) Healthy controls and patients with Parkinson’s disease included for DTI analysis as fractional anisotropy and mean diffusivity changes.

Here, we hypothesized that structural and microstructural changes in the cholinergic system nuclei and associated limbic pathways could be underlying cognitive impairment in patients with Parkinson’s disease, and moreover could predict the development of cognitive impairment. We sought to investigate this hypothesis by performing a cross-sectional baseline comparison of MRI data between patients with Parkinson’s disease with and without cognitive impairment, and a longitudinal 36-month comparison of MRI data between those Parkinson’s patients who remained cognitively intact and those who developed cognitive impairment (Fig. 1).

## Materials and methods

### Study participants

Data used for this paper were obtained from the Parkinson’s Progression Markers Initiative database (www.ppmi-info.org/data) in January 2017. We excluded subjects with a history of stroke or transient ischaemic attack. A total of 304 patients with Parkinson’s disease not on Parkinson’s medications (drug-naïve) and 167 healthy control subjects were identified. All patients underwent an initial screening visit followed by a baseline visit where demographics, family history, clinical characteristics, cognitive status and non-motor symptoms measurements were collected (Supplementary material).

### Cognitive assessments

Cognitive status was assessed at baseline and follow-up visits every 6 months (terminated at 36 months, or earlier if a patient developed cognitive impairment). Cognitive function was defined as two levels. Level 1 diagnosis was done on all patients at baseline and was determined based on Montreal Cognitive Assessment (MoCA) scores. Patients with MoCA ≥26 were screened as cognitively normal (PD-MoCA ≥26; *n* = 232), and patients with MoCA ≤25 were screened as with cognitive impairment (PD-MoCA ≤25; *n* = 72). Level 2 diagnosis was determined at follow-up visits with MoCA ≤25, self-reported issues in cognitive function, and at least two cognitive test scores (irrespective of test domain) greater than 1.5 standard deviations (SD) below mean healthy control age/education standardized scores as published previously (Weintraub *et al.*, 2015). At the end of the 36-month period, 35 patients with Parkinson’s disease satisfied level 2 diagnosis for cognitive impairment, whereas 197 patients did not.

### Image acquisition and processing

T_1_- and T_2_-weighted images were acquired by Philips (GE), or Siemens machines with either 1.5 T or 3 T strength. Diffusion tensor imaging (DTI) images were obtained with Siemens machines using a 2D single-shot echo planar imaging sequence with 3 T strength (Supplementary material). T_1_-weighted images were preprocessed using Statistical Parametric Mapping 12 (SPM, Wellcome Trust Centre for Neuroimaging, London, UK) implemented in MATLAB (The Math-Works, Natick, MA, USA) to allow for grey matter voxel-based morphometry analysis. Images were segmented and modulated into grey matter, white matter, CSF, bone and soft tissue. The grey matter image was smoothed to adjust for functional anatomical variability, and normalized and aligned into Montreal Neurological Institute (MNI) space. The final T_1_-weighted image map represents the volume of grey matter within each voxel. T_2_-weighted images were co-registered with T_1_-weighted for each subject to rule out vascular pathology and quantify the volume of white matter lesions.

Diffusion weighting was isotropically distributed along 64 gradient directions with a b-value of 1000 s/mm^2^, and a non-diffusion-weighted imaging (b0) was acquired at the start of each scan. Diffusion data analysis was performed with FSL Diffusion Toolbox (FMRIB Software Library, Centre Software Library, University of Oxford, Oxford, UK); topup (Andersson *et al.*, 2003) and eddycorrect (Andersson and Sotiropoulos, 2016) corrected for head motion, artefacts and eddy currents. DTIFit fitted diffusor tensor model maps at each voxel to generate fractional anisotropy maps, and mean diffusivity maps (Supplementary material).

Grey matter analysis was done by T_1_-weighted images obtained from all patients described (Fig. 1A). DTI analysis was done on a subset of 62 healthy controls and 84 patients with Parkinson’s disease (Fig. 1B). Of the patients with Parkinson’s disease, 64 were screened as not cognitively impaired at baseline (PD-MoCA ≥26) and 20 were screened as cognitively impaired at baseline (PD-MoCA ≤25). Patients not cognitively impaired at baseline screening were followed up for the same period: 17 patients with Parkinson’s disease satisfied level 2 diagnosis for cognitive impairment, whereas 47 patients did not.

To account for the variability across MRI scanners, we investigated differences in grey matter mean voxel values obtained by different MRI manufacturers (Philips versus GE versus Siemens) and with different strength of field (1.5 versus 3 T). We also investigated differences in DTI mean diffusivity mean voxel values obtained with different protocols (gated versus non-gated). Considering that the variability in DTI across cameras is usually high, we investigated the cross-centre variance of mean diffusivity mean voxel values.

### Region of interest analysis

**Figure 2:**
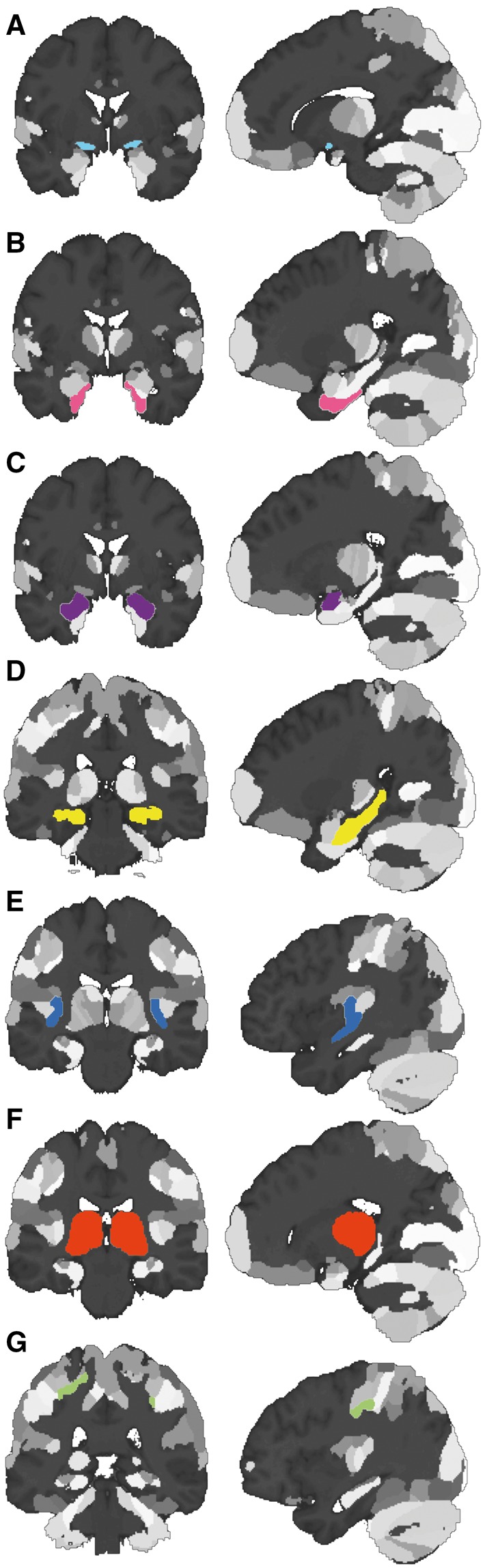
***A priori* regions of interest.** Anatomy Toolbox regions of interest in MNI space displayed in coronal (*left*) and sagittal (*right*) views. (**A**) Nucleus basalis of Meynert, (**B**) entorhinal cortex, (**C**) amygdala, (**D**) hippocampus, (**E**) insula, (**F**) thalamus, and (**G**) primary somatosensory cortex.

Regions of interest were identified using probabilistic anatomical maps available in SPM 12 Anatomy Toolbox (Eickhoff *et al.*, 2005). Probabilistic anatomical maps were created from microscopic histological post-mortem analysis of 10 brains. Each map describes the anatomical probability of finding the region of interest at each voxel in MNI reference space, based on the relative frequency of finding the areas in the same space in the 10-brain analysis. Based on the probability maps, each voxel was assigned to the most probable area by applying an algorithm to the probabilistic anatomical maps available in the toolbox, previously described (Eickhoff *et al.*, 2005) (Fig. 2). The nucleus basalis of Meynert is located in the basal forebrain, which is composed of cholinergic cell groups defined histologically as Ch1–Ch6. Ch4 corresponds to the nucleus basalis of Meynert (Mesulam *et al.*, 1983), and was identified using an existing available probabilistic anatomical map (Zaborszky *et al.*, 2008). Similarly, the entorhinal cortex, amygdala, hippocampus, and insula were identified using existing available probabilistic anatomical maps (Kurth *et al.*, 2010). The thalamus was identified using the Thalamic Connectivity atlas by Behrens *et al.* (2003). A reference region was identified as the primary somatosensory cortex area 3a through existing available probabilistic anatomical maps (Choi *et al.*, 2006). This area confirmed anatomical findings, as the primary somatosensory cortex is relatively unaffected in Parkinson’s disease and dementia pathology (Burton *et al.*, 2004). Region of interest analysis was performed on normalized MNI space images and repeated on co-registered T_1_-weighted applying the partial volume correction (Zhang *et al.*, 2016). Potential artefacts due to partial volume were reduced by extracting regions of interest in DTI conditioned on brain tissue content derived from the corresponding segmented structural MRI data. Specifically, to reduce artefacts due to brain atrophy, the regions of interest were extracted from regions with more than 90% probability of brain tissue content. To reduce artefacts to partial brain tissue volume further, a threshold of more than 50% probability of grey matter content was applied for regions of interest in grey matter areas, as previously done in other Parkinson’s Progression Markers Initiative (PPMI) studies (Zhang *et al.*, 2016).

### Voxel-based analysis

Voxel-wise statistics for between-group comparisons were computed using appropriately weighted contrasts to localize significant changes in mean voxel values on voxel-based morphometry and DTI fractional anisotropy and mean diffusivity MNI images. The contrasts were used to derive Z-scores on a voxel basis using the general linear model. The threshold for statistical significance was set to *P* < 0.001 (uncorrected). Voxel-based analysis was performed using SPM 12 implemented in MATLAB 8.4.

### White matter lesion volume

Quantification of white matter lesions was performed by using T_1_-weighted and T_2_/FLAIR MRI (Leritz *et al.*, 2014). White matter lesion volumes in T_1_-weighted were calculated with the Freesurfer image analysis suite (http://surfer.nmr.mgh.harvard.edu/), as described previously (Fischl *et al.*, 2002, 2004). Each subject’s T_1_-weighted lesion mask was overlaid on co-registered T_2_/FLAIR images for quality control on final volumetric data (Supplementary material). In detail, the volumetric T_1_-weighted images were processed to remove non-brain tissue using a hybrid watershed/surface deformation procedure (Segonne *et al.*, 2004), automated Talairach transformation, and segmentation of the subcortical white matter and deep grey matter volumetric structures (Fischl *et al.*, 2002, 2004). White matter lesions were labelled using a probabilistic procedure (Fischl *et al.*, 2002). Total white matter lesion (hypointensity) volume was then calculated for each hemisphere; these were averaged together to create a single white matter lesion volume for each subject. A manual quality check of the output of the Freesurfer analysis, for each individual MRI, was performed with the freeview software and lesions volume amended accordingly (G.P., J.B.). White matter lesion T_1_-hypointensity volumes are labelled in a more restricted portion of tissue compared to hyperintensity volumes measured on T_2_/FLAIR (Salat *et al.*, 2010). To reduce this bias, quality control was performed on final volumetric data by overlaying each subject’s lesion map on the T_2_/FLAIR image (J.S., G.P.). None of the lesion masks had errors that would require exclusion. There were minor errors, particularly in the determination of the boundaries of large lesions. However, we preferred not to correct them manually, as the intra-rater and inter-rater variability associated with manual delineations could potentially bias the results.

### Statistical analysis

Statistical analysis was performed in Statistical Package for Social Sciences (SPSS 23.0) software (SPSS Inc., Chicago, IL). Normality was tested with the Shapiro-Wilk test (<50 values) or Kolmogorov-Smirnov test (≥50 values), as appropriate. Continuous variables were expressed as mean and SD in parentheses and compared using independent samples *t*-test (normally distributed) and exact Mann-Whitney U-test (not normally distributed). Multivariate analysis of variance (MANOVA) was used to assess the main effects of regional structural and microstructural changes among the groups. If the overall multivariate test was significant, *P*-values for each variable were calculated following Bonferroni’s multiple comparisons test. Subsequently, MANOVA was repeated adding age as covariate. Categorical variables were expressed as proportions and compared using Fisher’s test. To determine predictors of cognitive decline, Cox survival proportional hazards analyses were performed using each region of interest as a predictor of cognitive impairment at univariate analysis. Multivariate Cox survival analyses were carried out including each significant region of interest at univariate and, as covariates, clinical and non-clinical predictors of cognitive impairment previously validated in the PPMI study by Schrag *et al.* (2017). The analyses have also been repeated including, as covariates in the model: age, white matter lesions volume and presence of axial motor symptoms. The first occurrence of cognitive impairment at follow-up was used as the time-to-event in the Cox model. To increase stability of our findings, we confirmed that the outcome present at one visit was still present at the subsequent visits. Grey matter mean voxel values were increased by a factor 100, while fractional anisotropy and mean diffusivity DTI mean voxel values were increased by a factor 1000 in Cox survival analysis. Kaplan–Meier survival estimates were generated after stratifying patients with Parkinson’s disease by abnormal region of interest mean voxel values compared to using log-rank (Mantel Cox) test. Abnormal region of interest mean voxel was defined as 1 SD from our population of healthy controls. A *P*-value < 0.05 was used as the cut-off point to be determined as a statistically significant result.

### Data availability

All data used in this study are available from the PPMI database (www.ppmi-info.org/data).

## Results

### Grey matter changes and cognitive impairment in patients with Parkinson’s disease

**Figure 3:**
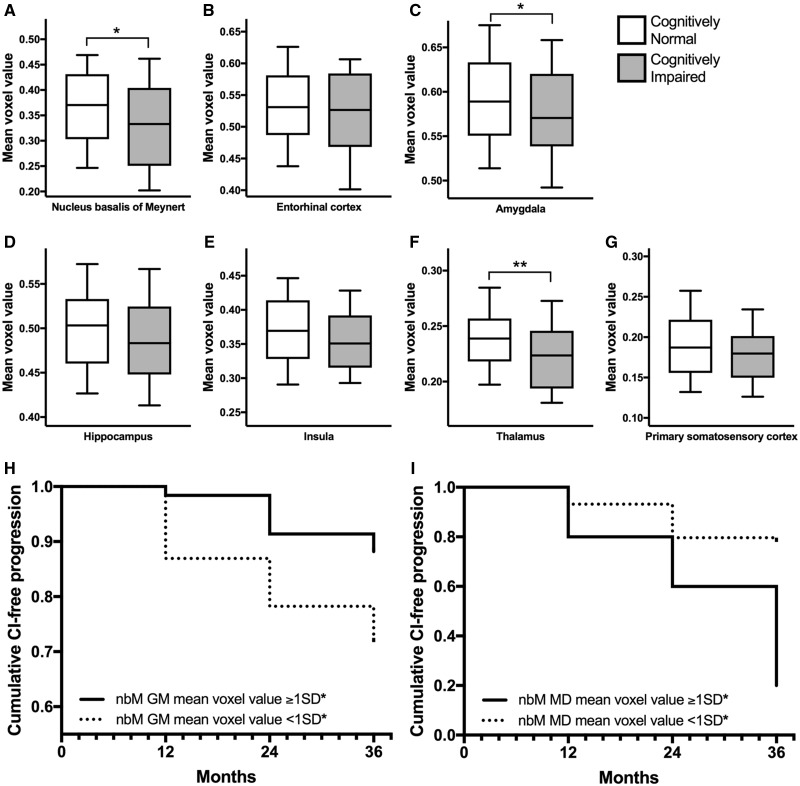
**Nucleus basalis of Meynert as a predictor of cognitive decline.** (**A**–**G**) Region of interest grey matter volume of patients with Parkinson’s disease screened at baseline for cognitive function. Patients stratified by cognitive function using MoCA test (level 1 diagnosis). Cognitively normal defined as MoCA ≥26 (*n* = 232), cognitively impaired defined as MoCA ≤25 (*n* = 72). Median, 10th, 25th, 75th and 90th percentile of region of interest mean voxel value shown as box plot. Groups compared by independent sample *t*-tests, with 302 degrees of freedom (equal variance assumed). Equality of variance tested by Levene’s test. Statistically significant results indicated by *P*-values: *P < 0.05, **P < 0.01. (**H**) Cumulative cognitive impairment-free (CI-free) progression amongst patients with Parkinson’s disease stratified by grey matter nucleus basalis of Meynert volume. Two hundred and thirty-two patients with Parkinson’s disease screened as cognitively normal at baseline. At follow-up for up to 36 months, 35 patients developed clinically confirmed CI (PD-CI) and 197 patients remained cognitively normal (PD-noCI). Patients stratified by a split of healthy control mean nucleus basalis of Meynert grey matter mean voxel values minus 1 SD (*) and Kaplan-Meier graph generated. Log-rank (Mantel-Cox) test indicates cumulative development of cognitive impairment over 36 months are statistically different (χ^2^* = *8.78, *P = *0.003, 1 degree of freedom). (**I**) Cumulative cognitive impairment-free (CI-free) progression amongst patients with Parkinson’s disease stratified by DTI mean diffusivity of the nucleus basalis of Meynert. Thirty-four patients with Parkinson’s disease screened as cognitively normal at baseline. At follow-up for up to 36 months, 17 patients developed clinically confirmed cognitive impairment (PD-CI) and 47 patients remained cognitively normal (PD-noCI). Patients were stratified by a split of healthy control mean nucleus basalis of Meynert mean diffusivity mean voxel values plus 1 SD (*) and Kaplan-Meier graph generated. Log-rank (Mantel-Cox) test indicates cumulative development of cognitive impairment over 36 months are statistically different (χ^2^* = *8.03, *P = *0.005, 1 degree of freedom). GM = grey matter; MD = mean diffusivity; nbM = nucleus basalis of Meynert.

A total of 304 patients with Parkinson’s disease were identified to be included in this study (Table 1). We first conducted a cross-sectional comparison of grey matter mean voxel values between healthy controls and patients with Parkinson’s disease at baseline. No differences were found for the nucleus basalis of Meynert, entorhinal cortex, amygdala, hippocampus, insula, thalamus, or primary somatosensory cortex (reference region). After partial volume correction, no differences were found in any regions of interest (Table 2). Then, we stratified patients with Parkinson’s disease into two subgroups: screened as cognitively normal (PD-MoCA ≥26, *n* = 232) and screened as cognitively impaired (PD-MoCA ≤25, *n* = 72). We found lower grey matter mean voxel values of nucleus basalis of Meynert (*P = *0.016), amygdala (*P = *0.022) and thalamus (*P = *0.001) in cognitively impaired versus cognitively normal patients with Parkinson’s disease (Fig. 3A–G). No differences were found for other regions of interest and the results were consistent when age was a covariate. After partial volume correction, we found lower grey matter mean voxel values of nucleus basalis of Meynert (*P = *0.01) and thalamus (*P < *0.001) but no differences in the amygdala or other areas (Table 3).

**Table 1:** Baseline demographics and clinical characteristics

	Study groups		
	Healthy controls	Parkinson’s disease patients	*t*-test
	Mean (SD)	Mean (SD)	
**Demographic features**			
Sex (female, male)	167 (57, 110)	304 (104, 200)	t = 0.02, *P = *0.99
Age	59.9 (11.4)	61.4 (9.5)	t = −1.49, *P = *0.14
Age of onset	−	60.9 (9.5)	−
Duration of disease	−	6.6 (6.6)	−
PD family history, positive %	4.3	25.3	t = −7.12, *P < *0.0001
Education	16.1 (2.8)	15.5 (2.9)	t = 2.02, *P = *0.04
**Clinical characteristics**			
MDS-UPDRS Part-I	0.6 (1.1)	1.2 (1.5)	t = −5.10, *P < *0.0001
MDS-UPDRS Part-I Quest.	2.4 (2.5)	4.3 (3.1)	t = −7.12, *P < *0.0001
MDS-UPDRS Part-II	0.4 (0.9)	5.9 (4.2)	t = −21.73, *P < *0.0001
MDS-UPDRS Part-III	1.3 (2.7)	20.9 (9.1)	t = −34.84, *P < *0.0001
Hoehn and Yahr scale	0.0 (0.1)	1.6 (0.5)	t = −50.79, *P < *0.0001
White matter volume	3107.3 (4837.3)	2956.9 (2988.8)	t = 0.42, *P* = 0.68
**Non-motor symptom status**			
Geriatric Depression Scale	1.4 (2.2)	2.3 (2.4)	t = −4.20, *P < *0.0001
Scales for Outcomes in Parkinson’s disease - Autonomic	5.7 (3.8)	9.5 (6.2)	t = −8.17, *P < *0.0001
Epworth Sleepiness Scale	5.7 (3.5)	5.7 (3.4)	t = −0.13, *P = *0.90
RBDSQ	2.9 (2.3)	4.2 (2.7)	t = −5.09, *P < *0.0001
UPSIT	34.1 (4.7)	22.3 (8.3)	t = 19.80, *P < *0.0001
**Cognitive status**			
MoCA	28.3 (1.1)	27.0 (2.2)	t = 7.84, *P < *0.0001
Semantic Fluency Test	51.6 (11.6)	48.2 (10.7)	t = 3.15, *P = *0.002
HVLT Immediate Recall	26.0 (4.5)	24.4 (5.0)	t = 3.42, *P = *0.001
Symbol Digit Modalities Test	47.6 (10.7)	41.7 (9.8)	t = 5.98, *P < *0.0001
Benton JLO	13.2 (2.0)	12.9 (2.1)	t = 1.77, *P = *0.008

Age, age of onset, and education measured in years, duration of disease measured in months. HVLT = Hopkin’s Learning Verbal Test; JLO = Benton Judgement of Line Orientation Test; PD = Parkinson’s disease.

**Table 2:** Baseline grey matter and DTI region of interest volumes between healthy controls and Parkinson’s disease patients

	Study groups (mean voxel value)		
	Healthy controls	Parkinson’s disease patients	*t*-test
	Mean (SD)	Mean (SD)	
**Grey matter region of interest volumes**			
Nucleus basalis of Meynert	0.361 (0.083)	0.357 (0.089)	t = 0.39, *P* = 0.69
Entorhinal cortex	0.530 (0.075)	0.526 (0.081)	t = 0.54, *P* = 0.59
Amygdala	0.587 (0.063)	0.587 (0.065)	t = −0.08, *P* = 0.94
Hippocampus	0.492 (0.055)	0.496 (0.059)	t = −0.78, *P* = 0.44
Insula	0.367 (0.056)	0.367 (0.057)	t = −0.09, *P* = 0.93
Thalamus	0.234 (0.035)	0.234 (0.034)	t = −0.04, *P* = 0.97
Primary somatosensory cortex	0.194 (0.050)	0.190 (0.048)	t = 0.77, *P* = 0.44
**Grey matter region of interest volumes after partial volume correction**			
Nucleus basalis of Meynert	0.397 (0.060)	0.392 (0.061)	t = 0.82, *P* = 0.41
Entorhinal cortex	0.515 (0.062)	0.514 (0.062)	t = 0.13, *P* = 0.90
Amygdala	0.514 (0.055)	0.514 (0.055)	t = 0.07, *P* = 0.95
Hippocampus	0.449 (0.053)	0.453 (0.053)	t = −0.70, *P* = 0.49
Insula	0.472 (0.057)	0.474 (0.054)	t = −0.31, *P* = 0.75
Thalamus	0.193 (0.028)	0.191 (0.027)	t = 0.70, *P* = 0.48
Primary somatosensory cortex	0.291 (0.053)	0.290 (0.050)	t = 0.16, *P* = 0.87
**DTI region of interest fractional anisotropy and mean diffusivity**			
**Fractional anisotropy**			
Nucleus basalis of Meynert	0.450 (0.035)	0.449 (0.034)	t = 0.13, *P* = 0.89
Entorhinal cortex	0.203 (0.017)	0.205 (0.019)	t = −0.64, *P* = 0.53
Amygdala	0.237 (0.018)	0.237 (0.019)	t = −0.03, *P* = 0.98
Hippocampus	0.209 (0.021)	0.210 (0.020)	t = −0.11, *P* = 0.91
Insula	0.223 (0.016)	0.222 (0.014)	t = 0.34, *P* = 0.73
Thalamus	0.313 (0.030)	0.312 (0.025)	t = 0.08, *P* = 0.94
Primary somatosensory cortex	0.151 (0.017)	0.152 (0.017)	t = −0.34, *P* = 0.73
**Mean diffusivity × 100**			
Nucleus basalis of Meynert	0.124 (0.016)	0.124 (0.015)	t = −0.18, *P* = 0.86
Entorhinal cortex	0.118 (0.020)	0.118 (0.014)	t = 0.10, *P* = 0.92
Amygdala	0.092 (0.009)	0.092 (0.009)	t = 0.14, *P* = 0.89
Hippocampus	0.113 (0.017)	0.115 (0.021)	t = −0.54, *P* = 0.59
Insula	0.106 (0.012)	0.108 (0.015)	t = −0.70, *P* = 0.49
Thalamus	0.096 (0.015)	0.096 (0.017)	t = 0.01, *P* = 0.99
Primary somatosensory cortex	0.105 (0.011)	0.105 (0.011)	t = −0.11, *P* = 0.92
**DTI region of interest fractional anisotropy and mean diffusivity after partial volume correction**			
**Fractional anisotropy**			
Nucleus basalis of Meynert	0.236 (0.052)	0.236 (0.051)	t = 0.05, *P* = 0.96
Entorhinal cortex	0.173 (0.024)	0.170 (0.019)	t = 0.69, *P* = 0.49
Amygdala	0.219 (0.031)	0.215 (0.025)	t = 0.72, *P* = 0.47
Hippocampus	0.253 (0.042)	0.250 (0.040)	t = 0.34, *P* = 0.73
Insula	0.220 (0.033)	0.221 (0.027)	t = −0.17, *P* = 0.86
Thalamus	0.369 (0.033)	0.373 (0.034)	t = −0.68, *P* = 0.49
Primary somatosensory cortex	0.081 (0.028)	0.084 (0.024)	t = −0.81, *P* = 0.42
**Mean diffusivity × 100**			
Nucleus basalis of Meynert	0.128 (0.027)	0.130 (0.030)	t = −0.32, *P* = 0.75
Entorhinal cortex	0.106 (0.013)	0.108 (0.012)	t = −1.12, *P* = 0.26
Amygdala	0.107 (0.012)	0.109 (0.012)	t = −1.05, *P* = 0.30
Hippocampus	0.112 (0.015)	0.112 (0.017)	t = −0.26, *P* = 0.79
Insula	0.108 (0.011)	0.112 (0.015)	t = −1.69, *P* = 0.09
Thalamus	0.123 (0.019)	0.123 (0.020)	t = 0.01, *P* = 0.99
Primary somatosensory cortex	0.079 (0.027)	0.084 (0.028)	t = −1.24, *P* = 0.22

**Table 3:** Baseline grey matter and DTI region of interest volumes between cognitively intact and cognitively impaired Parkinson’s disease patients

	Parkinson’s disease subgroups (mean voxel value)		
	Cognitively normal (PD-MoCA ≥26)	Cognitively impaired (PD-MoCA ≤25)	*t*-test
	Mean (SD)	Mean (SD)	
**Grey matter region of interest volumes Parkinson’s disease patients**			
Nucleus basalis of Meynert	0.364 (0.087)	0.335 (0.093)	t = 2.43, *P* = 0.02
Entorhinal cortex	0.530 (0.077)	0.515 (0.093)	t = 1.34, *P* = 0.18
Amygdala	0.592 (0.064)	0.572 (0.067)	t = 2.30, *P* = 0.02
Hippocampus	0.500 (0.056)	0.484 (0.065)	t = 1.98, *P* = 0.05
Insula	0.370 (0.057)	0.357 (0.056)	t = 1.79, *P* = 0.07
Thalamus	0.238 (0.032)	0.223 (0.036)	t = 3.23, *P* = 0.001
Primary somatosensory cortex	0.192 (0.048)	0.184 (0.047)	t = 1.31, *P* = 0.19
**Grey matter region of interest volumes in Parkinson’s disease patients after partial volume correction**			
Nucleus basalis of Meynert	0.396 (0.063)	0.377 (0.048)	t = 2.26, *P* = 0.01
Entorhinal cortex	0.518 (0.061)	0.503 (0.061)	t = 1.76, *P* = 0.08
Amygdala	0.517 (0.055)	0.502 (0.055)	t = 1.89, *P* = 0.06
Hippocampus	0.455 (0.052)	0.444 (0.055)	t = 1.61, *P* = 0.11
Insula	0.477 (0.055)	0.464 (0.052)	t = 1.65, *P* = 0.10
Thalamus	0.194 (0.026)	0.180 (0.028)	t = 3.74, *P* < 0.001
Primary somatosensory cortex	0.293 (0.049)	0.280 (0.051)	t = 1.96, *P* = 0.05
**DTI region of interest fractional anisotropy and mean diffusivity in Parkinson’s disease patients**			
**Fractional anisotropy**			
Nucleus basalis of Meynert	0.451 (0.034)	0.444 (0.035)	t = 0.83 *P* = 0.41
Entorhinal cortex	0.205 (0.020)	0.203 (0.017)	t = 0.52 *P* = 0.61
Amygdala	0.239 (0.020)	0.233 (0.017)	t = 1.07 *P* = 0.29
Hippocampus	0.210 (0.021)	0.207 (0.017)	t = 0.71 *P* = 0.48
Insula	0.224 (0.014)	0.219 (0.012)	t = 1.37 *P* = 0.18
Thalamus	0.314 (0.026)	0.307 (0.024)	t = 1.05 *P* = 0.30
Primary somatosensory cortex	0.153 (0.018)	0.149 (0.016)	t = 0.85 *P* = 0.40
**Mean diffusivity × 100**			
Nucleus basalis of Meynert	0.122 (0.013)	0.132 (0.019)	t = −2.30, *P* = 0.03
Entorhinal cortex	0.116 (0.013)	0.125 (0.016)	t = 0.48, *P* = 0.02
Amygdala	0.091 (0.007)	0.096 (0.013)	t = −1.60, *P* = 0.12
Hippocampus	0.111 (0.014)	0.127 (0.032)	t = −2.06, *P* = 0.05
Insula	0.106 (0.013)	0.115 (0.019)	t = −2.34, *P* = 0.02
Thalamus	0.093 (0.013)	0.106 (0.024)	t = −2.23, *P* = 0.04
Primary somatosensory cortex	0.104 (0.011)	0.109 (0.013)	t = −1.53, *P* = 0.13
**DTI region of interest fractional anisotropy and mean diffusivity in Parkinson’s disease patients after partial volume correction**			
**Fractional anisotropy**			
Nucleus basalis of Meynert	0.235 (0.048)	0.237 (0.061)	t = −0.16, *P* = 0.87
Entorhinal cortex	0.172 (0.019)	0.165 (0.020)	t = 1.36, *P* = 0.18
Amygdala	0.219 (0.023)	0.205 (0.028)	t = 2.17, *P* = 0.03
Hippocampus	0.256 (0.038)	0.234 (0.044)	t = 2.17, *P* = 0.03
Insula	0.218 (0.025)	0.231 (0.030)	t = −1.98, *P* = 0.05
Thalamus	0.379 (0.030)	0.354 (0.042)	t = 2.91, *P* = 0.005
Primary somatosensory cortex	0.083 (0.024)	0.088 (0.025)	t = −0.87, *P* = 0.38
**Mean diffusivity × 100**			
Nucleus basalis of Meynert	0.125 (0.026)	0.144 (0.037)	t = −2.11, *P* = 0.045
Entorhinal cortex	0.106 (0.011)	0.115 (0.015)	t = −3.20, *P* = 0.002
Amygdala	0.106 (0.007)	0.117 (0.018)	t = −2.52, *P* = 0.02
Hippocampus	0.109 (0.014)	0.124 (0.021)	t = −3.68, *P* < 0.001
Insula	0.110 (0.015)	0.115 (0.016)	t = −1.29, *P* = 0.20
Thalamus	0.119 (0.016)	0.135 (0.026)	t = −3.38, *P* = 0.001
Primary somatosensory cortex	0.082 (0.026)	0.091 (0.032)	t = −1.15, *P* = 0.25

**Figure 4:**
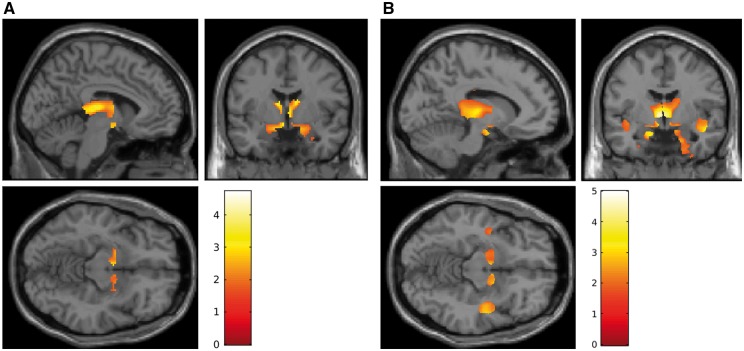
**Voxel-based analyses.** (**A**) Statistical parametric maps showing reduction in grey matter at voxel-based analysis cognitively impaired compared to cognitively normal patients with Parkinson’s disease (MNI co-ordinates nucleus basalis of Meynert, right: *x* = −4, *y* = −3, *z* = −9, left: *x* = −6, *y* = −1, *z* = −9; thalamus, right: *x* = 2, *y* = −13, *z* = 7, left: *x* = −2, *y* = −13, *z* = 7; and amygdala, right: *x* = −30, *y* = −111, *z* = −23, left: *x* = 30, *y* = −9, *z* = −23). Yellow–red areas represent voxel clusters with decreased values within the full brain. *P* < 0.001 uncorrected. The colour stripe indicates z-values. (**B**) Statistical parametric maps showing increase in mean diffusivity at voxel-based analysis in cognitively impaired compared to cognitively normal patients with Parkinson’s disease (MNI co-ordinates nucleus basalis of Meynert, right: *x* = −6, *y* = −4, *z* = −8, left: *x* = 6, *y* = −2, *z* = −7; thalamus, right: *x* = −5, *y* = −9, *z* = 1, left: *x* = 12, *y* = −21, *z* = 4; entorhinal cortex, right: *x* = −21, *y* = 4, *z* = −7, left: *x* = 23, *y* = 7, *z* = −26; and insula, right: *x* = −51, *y* = −17, *z* = −5, left: *x* = 48, *y* = −4, *z* = −16). Yellow–red areas represent voxel clusters with increase in mean diffusivity values within the full brain. *P* < 0.001 uncorrected. The colour stripe indicates z-values.

Region of interest analysis was repeated at voxel-based level and confirmed these results. Statistical parametric maps showed a reduction in grey matter at full brain voxel-based analysis in cognitively impaired compared to cognitively normal patients with Parkinson’s disease (Fig. 4A and Supplementary Table 1).

### Microstructural changes and cognitive impairment in patients with Parkinson’s disease

We conducted a cross-sectional comparison of DTI fractional anisotropy and mean diffusivity mean voxel values between healthy controls and patients with Parkinson’s disease on *a priori* region of interest selection at baseline. We found no differences in any fractional anisotropy or mean diffusivity region of interest mean voxel values. These results were confirmed at voxel-based level and after partial volume correction, which showed no differences in any areas (Table 2). Then, we stratified patients with Parkinson’s disease into two subgroups: screened as cognitively normal (PD-MoCA ≥26, *n* = 64) and screened as cognitively impaired (PD-MoCA ≤25, *n* = 20). We found increased mean diffusivity mean voxel values of nucleus basalis of Meynert (*P = *0.03), entorhinal cortex (*P = *0.02), insula (*P = *0.02), and thalamus (*P = *0.04) in cognitively impaired versus cognitively normal patients with Parkinson’s disease. No differences were found for other areas. After partial volume correction, we found higher mean diffusivity mean voxel values of the nucleus basalis of Meynert (*P* = 0.045), entorhinal cortex (*P* = 0.002), amygdala (*P* = 0.020), hippocampus (*P* < 0.001), and thalamus (*P* = 0.001) in cognitively impaired versus cognitively normal patients with Parkinson’s disease. We also found lower fractional anisotropy mean voxel values of the amygdala (*P* = 0.033), hippocampus (*P* = 0.033), and thalamus (*P* = 0.005) in cognitively impaired versus cognitively normal patients with Parkinson’s disease (Table 3).

Region of interest analysis was repeated at a voxel-based level and confirmed these results. Statistical parametric maps showed increased mean diffusivity in voxel-based analysis in cognitively impaired compared to cognitively normal patients with Parkinson’s disease (Fig. 4B and Supplementary Table 1).

### Grey matter changes as predictors of Parkinson’s disease cognitive decline

At multivariate Cox survival analysis of regions of interest and age as a covariate, the nucleus basalis of Meynert grey matter mean voxel value was the only statistically significant predictor of developing cognitive impairment (positive level 2 diagnosis) in Parkinson’s disease: hazard ratio (HR): 0.908, confidence interval (CI): 0.843–0.978, Wald: 6.435, *P = *0.011. After partial volume correction, the nucleus basalis of Meynert grey matter mean voxel value remained the only statistically significant predictor of developing cognitive impairment: HR: 0.906, CI: 0.830–0.991, Wald: 4.622, *P = *0.032 (Table 4).

**Table 4:** Grey matter and DTI predictors of cognitive impairment in Parkinson’s disease

	Hazard ratio	95% CI		Significance
		Lower	Upper	
**Grey matter predictors of cognitive impairment in Parkinson’s disease**				
Nucleus basalis of Meynert	0.908	0.843	0.978	*P* = 0.01
Entorhinal cortex	0.939	0.869	1.015	*P* = 0.12
Amygdala	1.000	0.871	1.148	*P* = 1.00
Hippocampus	1.081	0.933	1.251	*P* = 0.30
Insula	1.029	0.933	1.135	*P* = 0.57
Thalamus	1.011	0.893	1.146	*P* = 0.86
**Grey matter predictors of cognitive impairment in Parkinson’s disease after partial volume correction**				
Nucleus basalis of Meynert	0.907	0.830	0.991	*P* = 0.03
Entorhinal cortex	0.972	0.882	1.071	*P* = 0.56
Amygdala	1.055	0.885	1.259	*P* = 0.55
Hippocampus	1.051	0.884	1.249	*P* = 0.58
Insula	0.955	0.847	1.077	*P* = 0.45
Thalamus	1.012	0.850	1.204	*P* = 0.89
**DTI predictors of cognitive impairment in Parkinson’s disease**				
**Fractional anisotropy**				
Nucleus basalis of Meynert	1.003	0.985	1.021	*P* = 0.77
Entorhinal cortex	1.006	0.985	1.028	*P* = 0.56
Amygdala	1.002	0.951	1.056	*P* = 0.94
Hippocampus	0.986	0.958	1.014	*P* = 0.33
Insula	0.990	0.943	1.038	*P* = 0.67
Thalamus	1.010	0.982	1.039	*P* = 0.50
**Mean diffusivity**				
Nucleus basalis of Meynert	319.587	6.830	14 954.816	*P* = 0.003
Entorhinal cortex	0.015	0.001	0.942	*P* = 0.047
Amygdala	7.839	0.002	32 627.914	*P* = 0.63
Hippocampus	5.294	0.158	177.350	*P* = 0.35
Insula	17.892	0.257	1245.817	*P* = 0.18
Thalamus	1.042	0.013	84.864	*P* = 0.99
**DTI predictors of cognitive impairment in Parkinson’s disease after partial volume correction**				
**Fractional anisotropy**				
Nucleus basalis of Meynert	1.008	0.992	1.024	*P* = 0.33
Entorhinal cortex	1.017	0.977	1.058	*P* = 0.41
Amygdala	0.964	0.914	1.016	*P* = 0.17
Hippocampus	1.007	0.981	1.034	*P* = 0.59
Insula	1.003	0.987	1.019	*P* = 0.75
Thalamus	0.987	0.957	1.018	*P* = 0.40
**Mean diffusivity**				
Nucleus basalis of Meynert	11.371	1.025	126.172	*P* = 0.048
Entorhinal cortex	1.499	0.004	503.138	*P* = 0.89
Amygdala	3.554	<0.001	409.879	*P* = 0.83
Hippocampus	0.197	<0.001	87.934	*P* = 0.60
Insula	0.959	0.009	97.507	*P* = 0.99
Thalamus	1.250	0.002	842.332	*P* = 0.95

Cox survival proportional hazards analysis of grey matter region of interest mean voxel values. Cox survival up to 36 months follow-up of 232 Parkinson’s disease patients (screened at baseline as cognitively normal): 35 patients developed clinically confirmed cognitive impairment (PD-CI) and 197 patients remained cognitively normal (PD-noCI). Age is included as a covariate. Cox survival proportional hazards analysis of DTI fractional anisotropy and mean diffusivity region of interest mean voxel values. Cox survival (backwards: conditional) up to 36 months follow-up of 64 Parkinson’s disease patients (screened at baseline as cognitively normal): 17 patients developed clinically confirmed cognitive impairment (PD-CI) and 47 patients remained cognitively normal (PD-noCI). Hazard ratios produced with 95% CI and statistical significance.

Since previous studies have suggested that the University of Pennsylvania Smell Identification Test (UPSIT), REM Sleep Behaviour Disorder Screening Questionnaire (RBDSQ), Geriatric Depression Scale, Movement Disorder Society sponsored Unified Parkinson Disease Rating Scale Part-III (MDS-UPDRS-III), postural instability, *APOE* genotype group, and amyloid-β:tau ratio could also be predictors of cognitive impairment (Muller *et al.*, 2013; Schrag *et al.*, 2017), we carried out further multivariate Cox survival analyses to determine whether nucleus basalis of Meynert grey matter mean voxel value remained a statistically significant predictor of developing cognitive impairment when adjusting for each of these variables as covariates in addition to age. We found that nucleus basalis of Meynert grey matter mean voxel value remained a predictor of cognitive impairment when adjusted for UPSIT, RBDSQ, Geriatric Depression Scale, MDS-UPDRS-III, *APOE*, amyloid-β:tau, axial gait score, and white matter lesions volume (Supplementary Table 2). We then carried out a multivariate Cox survival analysis with all nine parameters as covariates and further confirmed the nucleus basalis of Meynert grey matter mean voxel value as a statistically significant predictor of developing cognitive impairment: HR: 0.919, CI: 0.870–0.971, Wald: 9.211, *P = *0.002. As previously indicated by Schrag *et al.* (2017), we also found UPSIT (HR: 0.945, CI: 0.894–0.999, Wald: 4.014, *P = *0.045) and amyloid-β:tau (HR: 202.716, CI: 5.870–7000.656, Wald: 8.640, *P = *0.003) to be significant predictors of cognitive impairment in the Cox survival analysis with all parameters as covariates. We further compared these parameters between the patient groups who developed cognitive impairment and those who did not. We found worse scores among patients who developed cognitive impairment in the UPSIT, RBDSQ, Geriatric Depression Scale, MDS-UPDRS-III, amyloid-β:tau ratio, and axial gait score. There were no differences in *APOE* genotype or white matter lesion volume (Supplementary Table 3).

Multivariate Cox survival with the same nine parameters was carried out after partial volume correction and showed the nucleus basalis of Meynert grey matter mean voxel value as a significant predictor of cognitive impairment: HR: 0.928, CI: 0.865–0.995, Wald: 4.436, *P = *0.035. Similarly, we also found the Geriatric Depression Scale (HR: 1.175, CI: 1.007–1.372, Wald: 4.189, *P = *0.041) and amyloid-β:tau (HR: 78.317, CI: 2.501–2452.692, Wald: 6.158, *P = *0.013) to be predictors of cognitive impairment.

We stratified patients using the average of nucleus basalis of Meynert grey matter mean voxel values within healthy controls minus 1 SD (0.278 mean voxel value) and generated Kaplan-Meier estimates (Fig. 3H). The groups had significantly different cognitive impairment-free survival times (χ^2^ = 8.78, *P = *0.003, 1 degree of freedom) when compared by a log-rank (Mantel-Cox) test.

### Microstructural changes as predictors of Parkinson’s disease cognitive decline

At multivariate Cox survival analysis of mean diffusivity region of interest, we found the nucleus basalis of Meynert mean voxel value (HR: 319.587, CI: 6.830–14954.816, Wald: 8.638, *P = *0.003) and entorhinal cortex (HR: 0.015, CI: 0.001–0.942, Wald: 3.953, *P = *0.047) mean voxel value to be statistically significant predictors of developing cognitive impairment (Table 4). We then carried out a multivariate Cox survival analysis with all nine parameters as covariates and further confirmed nucleus basalis of Meynert mean diffusivity mean voxel value as a statistically significant predictor of developing cognitive impairment (HR: 116.445, CI: 1.085–12497.762, Wald: 3.977, *P = *0.046). We carried out further bivariate Cox survival analysis to determine whether the nucleus basalis of Meynert mean diffusivity mean voxel value remained a statistically significant predictor of developing cognitive impairment when adjusting for each clinical variable and biomarkers as covariates. We found that the nucleus basalis of Meynert mean diffusivity mean voxel value remained a predictor of cognitive impairment when adjusted for the UPSIT, RBDSQ, Geriatric Depression Scale, MDS-UPDRS-III, *APOE*, amyloid-β:tau ratio, axial gait, and white matter lesion volume (Supplementary Table 2). After partial volume correction, at multivariate Cox survival analysis the nucleus basalis of Meynert mean diffusivity mean voxel value was the only significant predictor of cognitive impairment (HR: 11.371, CI: 1.025–126.172, Wald: 3.920, *P = *0.048) (Table 4). At multivariate Cox survival analysis of fractional anisotropy regions of interest, no areas were statistically significant predictors of developing cognitive impairment (positive level 2 diagnosis) in Parkinson’s disease.

We stratified patients using the healthy control mean nucleus basalis of Meynert DTI mean diffusivity mean voxel values plus 1 SD (0.00140 mean voxel value) and generated Kaplan-Meier estimates (Fig. 3I). The groups had significantly different cognitive impairment-free survival times (χ^2^ = 8.03, *P = *0.005, 1 degree of freedom) when compared by a log-rank (Mantel-Cox) test.

### Comparison of predictive value between grey and microstructural white matter data

To identify the better predictor between structural and microstructural changes within the nucleus basalis of Meynert, we performed a bivariate Cox survival including both mean diffusivity and grey matter nucleus basalis of Meynert. We found mean diffusivity to be a statistically significant predictor of cognitive impairment (HR: 72.73, CI: 1.916–2760.399, Wald: 5.338, *P = *0.021), but not grey matter (HR: 0.941, CI: 0.881–1.006, Wald: 3.200, *P = *0.074).

### Axial symptoms and cognitive impairment

We further investigated whether axial gait is associated with cognitive decline in our population of drug-naïve patients with Parkinson’s disease. In the cross-sectional baseline analysis, we found no difference in axial gait score (*P* = 0.21). However, at follow-up analysis, cognitively normal patients with Parkinson’s disease who developed cognitive impairment had a higher axial gait score (*P* = 0.02, Supplementary Table 3). These results were stable after including age as a covariate in the Cox survival analysis (HR: 3.144, CI: 1.007–9.810, Wald: 3.892, *P = *0.049). However, in a multivariate Cox survival analysis including the axial gait score with grey matter nucleus basalis of Meynert and all nine parameters as covariates, the axial gait score loses its power prediction (HR: 2.255, CI: 0.493–10.316, Wald: 1.099, *P* = 0.294).

### White matter lesion volume and cognitive impairment

We investigated whether white matter lesion volume is associated with cognitive decline. We found no difference in white matter lesion volume between healthy controls and patients with Parkinson’s disease (*t = *0.416, *P = *0.68). In cognitively impaired patients with Parkinson’s disease, we found a trend (not significant) of increased white matter lesion volume compared to cognitively normal patients (2815.4 ± 2983.7 in PD-MoCA ≥26 versus 3410.9 ± 2980.6 in PD-MoCA ≤25; *t = *−1.48, *P = *0.14). At follow-up, cognitively normal patients with Parkinson’s disease who developed cognitive impairment did have a trend (not significant) of higher white matter lesion volume (*t = *−1.51, *P* = 0.14, Supplementary Table 3) compared to patients who did not develop cognitive impairment. In a Cox survival analysis, we found white matter lesion volume to be a predictor of developing cognitive impairment in Parkinson’s disease (HR: 1.000, CI: 1.000–1.000, Wald: 6.643, *P = *0.01). However, when the Cox survival analysis was adjusted for age as a covariate, white matter lesion volume lost its power of prediction (HR: 1.000, CI: 1.000–1.000, Wald: 0.821, *P = *0.365).

### Assessment of variability between MRI scanners

No region of interest differences in grey matter mean voxel values were found between T_1_-weighted data obtained by different manufacturers (Philips versus GE versus Siemens) or strength of field (1.5 versus 3 T). No region of interest differences in mean diffusivity mean voxel values were found between DTI data obtained by different DTI protocols (gated versus non-gated), or acquired at different centres (Supplementary Tables 4 and 5).

## Discussion

In this study, we set out to prove a hypothesis of a significant relationship between the damage of the cholinergic system and the development of cognitive impairment in Parkinson’s disease, and demonstrated that microstructural damage of the nucleus basalis of Meynert underlines and predicts clinical onset of cognitive impairment in Parkinson’s disease.

We investigated structural and microstructural changes in several brain regions related to the cholinergic system and associated limbic pathways in cognitively impaired compared to cognitively intact patients with Parkinson’s disease, and relative to a group of age-matched healthy controls. We also followed-up cognitively intact patients with Parkinson’s disease for 36 months to identify early predictors of cognitive impairment. We used MoCA as a screening tool for cognitive impairment at baseline, and level 2 diagnosis also including self-reported issues in cognitive function, and impairment on at least two cognitive domains at follow-up. A more sensitive screening tool was beneficial at baseline to look for predictors of cognitive impairment in all patients with current cognitive impairment or in early developmental stages of cognitive impairment. Additionally, when looking for predictors of cognitive impairment in a longitudinal design, we wanted to ensure that predictors were identified before the onset of clinical cognitive impairment. Therefore, we aimed to exclude all patients who had early detectable stages of cognitive impairment using this more sensitive test.

Cross-sectional comparison between patients with Parkinson’s disease and healthy controls revealed no differences in grey matter or DTI mean diffusivity or fractional anisotropy. Separate subcortical volumetric and microstructural differences were, however, identified between cognitively normal and cognitively impaired patients with Parkinson’s disease. We found loss of grey matter and increased DTI mean diffusivity in the nucleus basalis of Meynert and thalamus of patients with Parkinson’s disease with cognitive impairment, which indicates damage in these structures. The involvement of the nucleus basalis of Meynert in cognitive decline in Parkinson’s disease has been described previously (Gratwicke *et al.*, 2015). Additionally, here, decreased thalamic volume was observed in patients with Parkinson’s disease with cognitive impairment, as suggested by Chen *et al.* (2016), who closely associated early cognitive decline in Parkinson’s disease with the atrophy of the thalamus. These findings demonstrate that changes in both structural (reduced grey matter voxel mean) and microstructural (increased mean diffusivity) levels in cholinergic structures underline cognitive impairment in patients with Parkinson’s disease. Our results were confirmed with region of interest and voxel-based analyses, and after correction for partial volume effects. Interestingly, we found that cognitively impaired patients also had microstructural changes in the entorhinal cortex (increased mean diffusivity), but no changes in structural grey matter voxel-based morphometry. The entorhinal cortex has prominent cholinergic innervation (Heys *et al.*, 2012), and histopathology has demonstrated that it is also affected early by tau pathology (Braak *et al.*, 2006).

Our longitudinal findings indicated that structural and microstructural changes in the nucleus basalis of Meynert were predictive for developing cognitive impairment in patients with Parkinson’s disease. Degeneration of the nucleus basalis of Meynert occurs before the onset of cognitive impairment, or while cognitive impairment is subclinical. Using Cox survival analysis, we established that the hazard ratio for developing cognitive impairment increases by 9.3% per every 0.01 decrease in grey matter mean voxel value in the nucleus basalis of Meynert. We also established that the hazard ratio for developing cognitive impairment increases by a factor of 11.4 per every 0.001 increase in DTI diffusivity mean voxel value in the nucleus basalis of Meynert. Several studies have suggested indicators of cognitive impairment in Parkinson’s disease, and age is widely recognized as a risk factor of cognitive impairment in both patients with Parkinson’s disease and in the general population (Williams-Gray *et al.*, 2013). After adjusting for age in addition to other clinical and non-clinical indicators of cognitive impairment suggested by Schrag *et al.* (2017), damage in the nucleus basalis of Meynert remained a statistically significant indicator of cognitive impairment. Thereby, we provide further evidence that the degeneration of the nucleus basalis of Meynert precedes and predicts the onset of cognitive impairment, independent to other clinical and non-clinical markers of Parkinson’s disease.

Moreover, our findings show that if both grey matter mean voxel value and mean diffusivity of nucleus basalis of Meynert were included in the same Cox model, mean diffusivity remained the only predictor of cognitive impairment. This suggests that microstructural changes in the nucleus basalis of Meynert may precede the structural damage of grey matter measured with voxel-based morphometry. This is in line with the finding that cognitively impaired patients at baseline had increased mean diffusivity but normal grey matter voxel mean within the entorhinal cortex, which is the area with greater connections with nucleus basalis of Meynert.

Our findings, however, do not indicate when the degeneration of the nucleus basalis of Meynert starts, and how the onset of degeneration relates to the onset of cognitive impairment. Braak *et al.* (2003) suggested that Lewy body accumulation of cholinergic neurons in the basal forebrain occurs at the same stage as the degeneration of dopaminergic neurons in the substantia nigra pars compacta. This is in line with the finding of Burciu *et al.* (2017), who found microstructural changes measured in the substantia nigra are key predictors of motor progression in Parkinson’s disease. Taken together, our study with that of Burciu *et al.* (2017), show it is possible that measuring the pathological processes at microstructural levels with DTI may be a reliable tool to predict the development of cognitive decline (measuring the damage of the nucleus basalis of Meynert) and motor progression (measuring the damage of the substantia nigra) in the early stages of Parkinson’s disease. Future studies need to clarify this combined predictive value of DTI in the same individuals.

Previous studies have also suggested that white matter lesions are associated with cortical cholinergic deafferentation in elderly subjects with leukoaraiosis (Bohnen *et al.*, 2009*b*). White matter lesions at the frontal horns are in close proximity to cholinergic axons that originate in the nucleus basalis of Meynert (Bohnen *et al.*, 2009*a*). Therefore, these lesions may result in more significant cortical deafferentation because of the more proximal axonal disruption (Bohnen *et al.*, 2009*a*). We investigated the presence of white matter lesions (combining T_1_ and T_2_-weighted images) and found no differences in cognitively impaired compared to cognitively normal patients with Parkinson’s disease at baseline. In our population, white matter lesions longitudinally predicted the development of cognitive impairment. However, when corrected for age, white matter lesions lost their power of prediction, which suggests that the ageing process more than white matter lesions are associated with cognitive decline. White matter lesions have also been associated with gait dysfunction, another symptom of Parkinson’s disease probably related to the damage of cholinergic system (Bohnen and Albin, 2011*b*). The presence of gait dysfunction and the degree of axial symptoms have been associated with the development of cognitive impairment in Parkinson’s disease (Bohnen *et al.*, 2009*a*; Bohnen and Albin, 2011*b*; Muller *et al.*, 2013). We investigated the degree of axial involvement in our population of patients with early Parkinson’s disease. We found that cognitively impaired patients had greater axial gait symptoms compared to cognitively normal patients with Parkinson’s disease at baseline. Axial gait symptoms were predictive of cognitive decline when considered alone, but their power as predictors was lower than DTI or voxel-based morphometry. Moreover, in the full prognostic model including all the known predictors of cognitive impairment, axial gait symptoms lost their power of prediction. This may suggest that, in Parkinson’s disease patients with axial symptoms, the degeneration of cholinergic nuclei involves not only the pedunculopontine nucleus (associated with gait dysfunction) but also the nucleus basalis of Meynert. Thus, damage to the nucleus basalis of Meynert might be a common mediator of the development of cognitive impairment, explaining why most studies (that did not measure the damage of nucleus basalis of Meynert) found an association between gait and cognitive decline.

Our research provides a realistic, cost-effective and non-invasive way to identify patients with Parkinson’s disease at higher risk to develop cognitive impairment, before clinical symptomatic onset. This represents an unmet need: the opportunity to evaluate only one reliable predictor of cognitive impairment in common clinical practice to allow us to assess patients and stratify their risk of prodromal cognitive impairment at early stages of the disease, improving patient care and outcomes (Anang *et al.*, 2017). We provide a clinical tool to screen people in a routine clinical MRI. We propose that this can further be used by clinicians to assess sub-phenotypes of Parkinson’s disease at higher risk of cognitive impairment as well as to investigate cognitive impairment and non-motor symptom progression. Clinical trials may then be tailored on patients at high risk of cognitive impairment, increasing the power of the analysis for the identification of disease modification treatments.

In identifying regions of interest we used combined probabilistic maps available in Anatomy Toolbox, a relatively new method. We believe Anatomy Toolbox serves to provide more accurate region of interest parameters than conventional brain atlases without having to manually identify regions and correct for errors. Probabilistic maps account for individuals’ structural differences whilst utilizing a standardized mapping format. Additionally, we acknowledged a limitation of this study to be the difficulty in identifying cognitive impairment as a prodromal form of Parkinson’s disease dementia, rather than another form of dementia. To account for this, we distinguished between developing dementia after Parkinson’s disease, concurrent Parkinson’s disease and dementia, and dementia preceding Parkinson’s disease by excluding patients screened as cognitively impaired at baseline for follow-up study. This removed patients with dementia preceding Parkinson’s disease, and Parkinson’s disease and dementia concurrently, which may be caused by dementia with Lewy bodies (Bohnen and Albin, 2011*a*). However, the only conclusive way of confirming cognitive impairment in Parkinson’s disease as a prodromal stage of dementia is with a histological post-mortem exam.

In conclusion, we demonstrated here that (i) the nucleus basalis of Meynert is a predictor of cognitive impairment in a population of early drug-naïve Parkinson’s patients; (ii) microstructural changes are stronger predictors compared to structural changes, even after partial volume correction; (iii) structural changes at voxel-based morphometry and microstructural changes at DTI are significant predictors of cognitive decline also in a model including all the previously suggested predictors (from other studies) of cognitive impairment in Parkinson’s disease; and (iv) white matter lesions are predictors of cognitive impairment only when ageing in not accounted. As the prevalence of Parkinson’s disease increases exponentially with age and prevalence of cognitive impairment increases alongside with the evolution of Parkinson’s disease, a reliable biomarker to identify those patients at higher risk for cognitive impairment is now more important than ever.

## Funding

Data used in the preparation of this article were obtained from the Parkinson’s Progression Markers Initiative database (www.ppmi-info.org/data). For up-to-date information on the study, visit www.ppmi-info.org. Parkinson’s Progression Markers Initiative - a public-private partnership - is sponsored by the Michael J. Fox Foundation for Parkinson's Research (MJFF) and is co-funded by MJFF, Abbvie, Avid Radiopharmaceuticals, Biogen Idec, Bristol-Myers Squibb, Covance, Eli Lilly & Co., F. Hoffman-La Roche, Ltd., GE Healthcare, Genentech, GlaxoSmithKline, Lundbeck, Merck, MesoScale, Piramal, Pfizer and UCB. Industry partners are contributing to Parkinson’s Progression Markers Initiative through financial and in-kind donations and are playing a lead role in providing feedback on study parameters through the Industry Scientific Advisory Board (ISAB). Through close interaction with the study, the Industry Scientific Advisory Board is positioned to inform the selection and review of potential progression markers that could be used in clinical testing. M.P.'s research is supported by Parkinson's UK, Lily and Edmond J. Safra Foundation, the European Union FP-7 scheme, CHDI Foundation, Michael J Fox Foundation (MJFF) for Parkinson's research, and King's College London's NIHR Mental Health Biomedical Research Centre at South London and Maudsley NHS Foundation. G.P.'s research is supported by Lily and Edmond J. Safra Foundation. J.S., H.W., and J.A.F.B. report no funding or disclosures.

## Supplementary material

Supplementary material is available at *Brain* online.

## Supplementary Material

Supplementary Material

## Abbreviations

DTI: diffusion tensor imaging
MDS-UPDRS: Movement Disorder Society sponsored Unified Parkinson Disease Rating Scale Part-III
MoCA: Montreal Cognitive Assessment
RBDSQ: REM Sleep Behaviour Disorder Screening Questionnaire
UPSIT: University of Pennsylvania Smell Identification Test
