# Supplementary material for: Nucleus basalis of Meynert degeneration precedes and predicts cognitive impairment in Parkinson’s disease
Source: Brain. 2018 Mar 21;141(5):1501–16. doi: 10.1093/brain/awy072 (PMC6171218; doi:10.1093/brain/awy072)
Supplement: Supplementary Material [file awy072_supplementary_material.pdf]

Supplementary Material

Nucleus basalis of Meynert degeneration precedes and predicts cognitive impairment in Parkinson's disease

Jonathan Schulz[1]†, Gennaro Pagano[1]†, Juan Alberto Bonfante[1], Heather Wilson[1], and Marios Politis[1]*

Affiliations

[1]Neurodegeneration Imaging Group, Institute of Psychiatry, Psychology and Neuroscience, King's College London, London, UK

†These authors contributed equally to this work.

***Correspondence:**

Professor Marios Politis

Neurodegeneration Imaging Group

Maurice Wohl Clinical Neuroscience Institute, Ground Floor, G.35

Institute of Psychiatry, Psychology & Neuroscience (IoPPN)

125 Coldharbour Lane, Camberwell, London, SE5 9NU

Email: marios.politis@kcl.ac.uk

Clinical Assessments. Information collected from study participants included age, gender, family history of Parkinson's disease (including biological mother, biological father, full sibling, half sibling, maternal grandparents, paternal grandparents, maternal aunts/uncles, paternal aunts/uncles and children) and years of education. Age of onset and duration of disease information was also collected for patients diagnosed with Parkinson's disease. Parkinson's disease stage and severity was assessed using Hoehn & Yahr scale and Movement Disorder Society sponsored Unified Parkinson Disease Rating Scale (Part-II and Part-III assess motor burden, while Part-I and Part-I Questionnaire assess global non-motor symptom burden). The severity of axial symptoms (postural instability and gait dysfunction) was calculated using specific Movement Disorder Society sponsored Unified Parkinson Disease Rating Scale sub-items (sum of item 3.10 gait, item 3.11 freezing of gait and item 3.12 postural stability) (Pagano *et al.*, 2016). Non-motor symptoms were evaluated using specific tests. Geriatric Depression Scale (15-item) assessed depression, Scales for Outcomes in Parkinson's disease - Autonomic assessed autonomic dysfunction, Epworth Sleepiness Scale assessed excessive daytime sleepiness, Rapid Eye Movement Sleep Behaviour Disorder Screening Questionnaire assessed Rapid Eye Movement Sleep Behaviour Disorder, and University of Pennsylvania smell identification test assessed olfactory dysfunction. Cognitive status was assessed using the Montreal Cognitive Assessment (MoCA) and four further neuropsychiatric assessments: Semantic Fluency Test, Hopkin's Learning Verbal Test Immediate Recall, Symbol Digit Modalities Test, and Benton Judgement of Line Orientation. Apo-E genotype group and Amyloid-β:Tau ratio biospecimen data of Parkinson's disease patients was also downloaded from Parkinson's Progression Markers Initiative.

Imaging acquisition parameters. Philips Medical Systems, GE Medical Systems, and SIEMENS machines were used for the magnetic resonance imaging acquisition.

T1images were obtained for 55 subjects using Philips Medical Systems machines with the following protocols: matrix x=256-288, y=256-288, z=136-180; TE: 3.2-4.6ms; TR: 6.9-25ms; slice thickness 1.2-1.6mm (30 were acquired using 1.5T machines and 25 using 3T machines); for 118 subjects using GE Medical Systems machines with the following protocols: matrix x=256-512, y=256-512, z=34-248, TE: 1.4-9ms, TR: 5.8-350ms, slice thickness: 1.2-4mm (75 of these images were acquired using 1.5T machines and 43 using 3T machines); and for 299 subjects using SIEMENS machines with the following protocols: matrix x=240-512, y=256-512, z=80-167; TE: 3.0-3.9ms; TR: 1980-2300ms; slice thickness: 1-2mm (39 of these images were acquired using 1.5T machines and 260 using 3T machines).

T2 images were obtained with a 2D Axial Turbo Spin echo sequence (Pulse Sequence=SE/IR; flip angle: 90°; voxel resolution 0.9 x 0.9 x 3 mm^3; TE: 11ms; TR=8002.0 ms).

Diffusion tensor imaging images were obtained using SIEMENS machines with a 2D single-shot echo planar imaging sequence (flip angle: 90°; gradient directions=64; voxel resolution 2 x 2 x 2 mm^3; TE: 88ms). For the majority of the patients (132/146) diffusion tensor imaging images were obtained using a gated protocol (TE=9100). For 14 subjects, diffusion tensor imaging images were obtained using non-gated protocol (TE=900).

Imaging processing methods. Fractional anisotropy images were aligned into a common space using the nonlinear registration tool FNIRT (Andersson, 2017), which uses a b-spline representation of the registration warp field (Rueckert *et al.*, 1999). Next, the mean fractional anisotropy image was created and thinned to create a mean fractional anisotropy skeleton that represents the centres of all fibre tracts common to the entire sample. The mean skeleton was masked to only display voxels with fractional anisotropy values greater than 0.2 so as to avoid inclusion of regions that could be composed of multiple tissue types or fibre orientations. Each subject's aligned, common-space fractional anisotropy data were then

projected onto this skeleton to create a 4D skeletonized volume; this was then fed into voxel-wise group statistics. Data along the skeleton were smoothed utilizing an anatomical constraint to limit the smoothing to neighbouring data within adjacent voxels along the skeleton. The exact transformations derived for the fractional anisotropy maps were then applied to the mean diffusivity volumes for matched processing of image volumes per subject. Fractional anisotropy and mean diffusivity images were then co-registered to the T1-weighted images. For regions-of-interest analysis, fractional anisotropy and mean diffusivity values were derived from Montreal Neurological Institute space and from each participant's native space. To increase reliability of our findings (Henf *et al.*, 2018), partial volume correction was used to account for the effect of cerebrospinal fluid in grey matter voxels, as performed previously on Parkinson's Progression Markers Initiative data (Zhang *et al.*, 2016). Potential artefacts due to partial volume were reduced by extracting regions-of-interests in diffusion tensor imaging conditioned on brain tissue content derived from the corresponding segmented structural magnetic resonance imaging data. Specifically, to reduce artefacts due to brain atrophy, the regions-of-interests were extracted from regions with more than 90% probability of brain tissue content. To further reduce artefacts to partial brain tissue volume, a threshold of more than 50% probability of grey matter content was applied for regions-of-interests in grey matter areas (Zhang *et al.*, 2016). The fractional anisotropy map represents microstructural integrity within each voxel, by analysing diffusion directionality, and the mean diffusivity map represents diffusivity within each voxel, by analysing the average molecular motion. T2-weighted fluid attenuated inversion recovery (FLAIR) sequence were used to rule out pathology or vascular changes. FLAIR images were further used to quantify the volume of white matter lesions. Full data acquisition procedures are described in the Supplementary materials and available online at www.ppmi-info.org.

White matter lesion volume. Quantification of white matter lesions was performed by using T1-weighted and T2/FLAIR magnetic resonance imaging (Leritz *et al.*, 2014). White matter lesions volumes in T1-weighted were calculated with the Freesurfer image analysis suite (http://surfer.nmr.mgh.harvard.edu/), as described previously (Fischl *et al.*, 2002; Fischl *et al.*, 2004). Each subject's T1-weighted lesion mask was overlaid on T2/FLAIR images for quality control on final volumetric data. In details, the volumetric T1-weighted images were processed to remove non-brain tissue using a hybrid watershed/surface deformation procedure (Segonne *et al.*, 2004), automated Talairach transformation, and segmentation of the subcortical white matter and deep grey matter volumetric structures (Fischl *et al.*, 2002; Fischl *et al.*, 2004). Freesurfer morphometric procedures have been demonstrated to show good test-retest reliability across scanner manufacturers and across field strengths (Han *et al.*, 2006). In addition, the automated segmentation procedures have demonstrated comparable reliability and validity when compared to manual procedures (Fischl *et al.*, 2002). White matter lesions were labelled using a probabilistic procedure (Fischl *et al.*, 2002). Total white matter lesions (hypointensity) volume was then calculated for each hemisphere; these were averaged together to create a single white matter lesions volume for each subject. A manual quality check of the output of the Freesurfer analysis, for each individual magnetic resonance imaging, was performed with the freeview software and lesions volume amended accordingly (G.P., J.B.). White matter lesions T1-hypointensity to be labelled in a more restricted portion of tissue compared to hyperintensity volumes measured on T2/FLAIR (Salat *et al.*, 2010). To reduce this bias, quality control was performed on final volumetric data by overlaying each subject's lesion map on the FLAIR image (J.S., G.P.). None of the lesion masks had errors that would require exclusion. There were minor errors, particularly in the determination of the boundaries of large lesions.

However, we did not prefer to correct them manually, as the intra-rater and inter-rater variability associated with manual delineations could potentially bias the results.

Supplementary Table 1. Voxel based analyses

	**Voxel based morphometry statistical parametric maps**				
**Montreal Neurological Institute co-ordinates**					
x	*y*	*z*		**Area**	**P-value**
-4	-3	-9	*Right*	Nucleus basalis of Meynert	*P*<0.001
6	-1	-9	*Left*		*P*<0.001
-2	-13	7	*Right*	Thalamus	*P*<0.001
2	-13	7	*Left*		*P*<0.001
-30	-11	-23	*Right*	Amygdala	*P*<0.001
30	-9	-23	*Left*		*P*<0.001
	**Statistical parametric maps of mean diffusivity images**				
**Montreal Neurological Institute co-ordinates**					
x	*y*	*z*		**Area**	**P-value**
-6	-4	-8	*Right*	Nucleus basalis of Meynert	*P*<0.001
6	-2	-7	*Left*		*P*<0.001
-5	-9	1	*Right*	Thalamus	*P*<0.001
12	-21	4	*Left*		*P*<0.001
-21	4	-27	*Right*	Entorhinal cortex	*P*<0.001
23	7	-26	*Left*		*P*<0.001
-51	-17	5	*Right*	Insula	*P*<0.001
48	-4	-16	*Left*		*P*<0.001

Contrast: -1 1; Height threshold: *P*<0.001; Extent threshold: 10 voxels.

Supplementary Table 2. Power of prediction cognitive impairment of nucleus basalis of Meynert in a bivariate with each clinical predictor of cognitive decline

**Grey matter regions-of-interest volumes**				
	**Hazard ratio**	**95% confidence interval**	**Wald**	**Significance**
University of Pennsylvania smell identification test	0.944	0.905–0.984	7.257	P=0.007
Rapid Eye Movement Sleep Behaviour Disorder Screening Questionnaire	0.934	0.903–0.984	7.12	P=0.008
Geriatric Depression Scale	0.957	0.918–0.998	4.287	P=0.038
MDS-UPDRS Part-III	0.957	0.918–0.998	4.276	P=0.039
Apo-E	0.936	0.893–0.982	7.214	P=0.007
Amyloid-ß:Tau	0.952	0.914–0.992	5.542	P=0.019
Axial gait score	0.952	0.913–0.992	5.54	P=0.019
White matter lesions volume	0.955	0.916–0.996	4.63	P=0.031
**Diffusion tensor imaging mean diffusivity**				
	**Hazard ratio**	**95% confidence interval**	**Wald**	**Significance**
University of Pennsylvania smell identification test	58.723	1.314–2625.30	4.413	P=0.036
Rapid Eye Movement Sleep Behaviour Disorder Screening Questionnaire	213.675	3.923–11637.1	6.918	P=0.009
Geriatric Depression Scale	192.179	4.531–8150.956	7.564	P=0.006
MDS-UPDRS Part-III	53.577	1.338–2145.998	4.471	P=0.034
Apo-E	128.287	2.763–5956.071	6.146	P=0.013
Amyloid-ß:Tau	51.876	1.155–2329.81	4.138	P=0.042
Axial gait score	311.63	6.780–14323.61	8.643	P=0.003
White matter lesions volume	84.774	1.884–3815.534	5.225	P=0.031

MDS-UPDRS: Movement Disorder Society sponsored Unified Parkinson Disease Rating Scale (Part-I, Part-I Questionnaire, Part-II, and Part-III). Hazard ratios produced with 95% confidence interval and statistical significance.

Supplementary Table 3. Proposed predictors of cognitive impairment in Parkinson's disease patients

	**Parkinson's disease subgroups (mean voxel value)**		
	**Did not develop cognitive impairment (PD-noCI)**	**Developed cognitive impairment (PD-CI)**	**t-test**
University of Pennsylvania smell identification test	23.45 (7.98)	18.94 (9.18)	t=3.00 P=0.003
Rapid Eye Movement Sleep Behaviour Disorder Screening	4.02 (2.59)	5.06 (2.82)	t=-2.14 P=0.03
Geriatric Depression Scale	2.12 (2.31)	3.11 (2.49)	t=-2.31 P=0.02
MDS-UPDRS Part-III	19.42 (8.58)	24.03 (8.56)	t=-2.92 P=0.004
Apo-E	0.01 (0.90)	0.07 (1.08)	t=-0.30 P=0.76
Amyloid-β:Tau	0.11 (0.05)	0.18 (0.11)	t=-3.17 P=0.003
Axial gait score	0.20 (0.22)	0.30 (0.30)	t=0.13 P=0.02
White matter lesions volume	2606.65 (2303.36)	3984.37 (5304.23)	t=-1.51 P=0.14

Parkinson's disease patients screened as cognitively normal at baseline and did not develop cognitive impairment within 36 months (PD-noCI, n=197) and Parkinson's disease patients screened as cognitively normal at baseline and developed cognitive impairment within 36 months (PD-CI, n=35). Tabled values are the mean voxel value of each regions-of-interest with standard deviation in parenthesis. MDS-UPDRS: Movement Disorder Society sponsored Unified Parkinson Disease Rating Scale.

Supplementary Table 4 | Cross-protocol variance of diffusion tensor imaging

	**Study groups (mean voxel value)**		
	**Diffusion tensor imaging gated**	**Diffusion tensor imaging non-gated**	**t-test**
Fractional anisotropy			
Nucleus basalis of Meynert	0.451 (0.035)	0.440 (0.035)	t=-1.09 P=0.28
Entorhinal cortex	0.204 (0.019)	0.205 (0.013)	t=0.21 P=0.83
Amygdala	0.237 (0.019)	0.243 (0.016)	t=1.15 P=0.25
Hippocampus	0.209 (0.021)	0.216 (0.014)	t=1.31 P=0.19
Insula	0.223 (0.015)	0.225 (0.010)	t=0.61 P=0.54
Thalamus	0.311 (0.028)	0.322 (0.020)	t=1.43 P=0.15
Primary somatosensory cortex	0.082 (0.026)	0.091 (0.032)	t=-1.15 P=0.25
Mean diffusivity*100			
Nucleus basalis of Meynert	0.124 (0.015)	0.125 (0.016)	t=0.22 P=0.83
Entorhinal cortex	0.119 (0.017)	0.111 (0.015)	t=-1.62 P=0.11
Amygdala	0.092 (0.009)	0.092 (0.008)	t=-0.14 P=0.89
Hippocampus	0.114 (0.020)	0.113 (0.012)	t=-0.18 P=0.86
Insula	0.107 (0.014)	0.109 (0.012)	t=0.56 P=0.57
Thalamus	0.096 (0.016)	0.099 (0.016)	t=0.64 P=0.53
Primary somatosensory cortex	0.079 (0.027)	0.084 (0.028)	t=-1.24 P=0.22

Tabled values are the mean voxel value of each regions-of-interest with standard deviation in parenthesis.

Supplementary Table 5. Cross-centre variance of diffusion tensor imaging mean diffusivity within Nucleus basalis of Meynert

Centre	Heathy controls			Parkinson's disease patients		
	**Number of patients**	**Mean diffusivity**	**CoV**	**Number of patients**	**Mean diffusivity**	**CoV**
1	2	0.142 (0.004)	0.028	10	0.125 (0.013)	0.104
2	4	0.125 (0.015)	0.120	7	0.126 (0.017)	0.135
3	5	0.127 (0.008)	0.063	5	0.120 (0.014)	0.117
4	10	0.121 (0.013)	0.107	9	0.124 (0.018)	0.145
5	3	0.117 (0.010)	0.085	5	0.115 (0.004)	0.035
6	8	0.121 (0.014)	0.116	8	0.122 (0.016)	0.131
7	5	0.125 (0.015)	0.120	11	0.124 (0.021)	0.169
8	3	0.142 (0.034)	0.239	1	0.120 (-)	-
9	7	0.119 (0.017)	0.143	11	0.129 (0.017)	0.132
10	8	0.130 (0.016)	0.123	12	0.130 (0.015)	0.115
11	6	0.117 (0.018)	0.154	3	0.118 (0.003)	0.025
12	1	0.105 (-)	-	2	0.115 (0.007)	0.061
ANOVA		*P*=0.35			*P*=0.84	
**After partial volume correction**						
**Centre**	**Heathy controls**			**Parkinson's disease patients**		
	**Number of patients**	**Mean diffusivity**	**CoV**	**Number of patients**	**Mean diffusivity**	**CoV**
1	2	0.145 (0.024)	0.025	10	0.149 (0.021)	0.102
2	4	0.120 (0.018)	0.118	7	0.122 (0.038)	0.133
3	5	0.125 (0.030)	0.060	5	0.110 (0.031)	0.114
4	10	0.125 (0.027)	0.112	9	0.123 (0.025)	0.142
5	3	0.115 (0.018)	0.089	5	0.119 (0.009)	0.031
6	8	0.136 (0.024)	0.114	8	0.136 (0.037)	0.130
7	5	0.141 (0.023)	0.117	11	0.124 (0.027)	0.166
8	3	0.158 (0.045)	0.240	1	0.102 (-)	-
9	7	0.115 (0.028)	0.140	11	0.132 (0.034)	0.128
10	8	0.134 (0.027)	0.123	12	0.144 (0.030)	0.114
11	6	0.120 (0.026)	0.157	3	0.118 (0.002)	0.024
12	1	0.099 (-)	-	2	0.114 (0.012)	0.061
ANOVA		*P*=0.45			*P*=0.25	

Tabled values are the mean voxel value of Nucleus basalis of Meynert mean diffusivity with standard deviation in parenthesis. CoV: Coefficient of variability.

References

Andersson JJ, M.; Smith, S. Non-linear registration aka Spatial normalisation: FMRIB Centre, Oxford, United Kingdom; 2017.

Fischl B, Salat DH, Busa E, Albert M, Dieterich M, Haselgrove C, *et al.* Whole brain segmentation: automated labeling of neuroanatomical structures in the human brain. Neuron 2002; 33(3): 341-55.

Fischl B, Salat DH, van der Kouwe AJ, Makris N, Segonne F, Quinn BT, *et al.* Sequence-independent segmentation of magnetic resonance images. NeuroImage 2004; 23 Suppl 1: S69-84.

Han X, Jovicich J, Salat D, van der Kouwe A, Quinn B, Czanner S, *et al.* Reliability of MRI-derived measurements of human cerebral cortical thickness: the effects of field strength, scanner upgrade and manufacturer. NeuroImage 2006; 32(1): 180-94.

Henf J, Grothe MJ, Brueggen K, Teipel S, Dyrba M. Mean diffusivity in cortical gray matter in Alzheimer's disease: The importance of partial volume correction. Neuroimage Clin 2018; 17: 579-86.

Leritz EC, Shepel J, Williams VJ, Lipsitz LA, McGlinchey RE, Milberg WP, *et al.* Associations between T1 white matter lesion volume and regional white matter microstructure in aging. Human brain mapping 2014; 35(3): 1085-100.

Pagano G, Ferrara N, Brooks DJ, Pavese N. Age at onset and Parkinson disease phenotype. Neurology 2016; 86(15): 1400-7.

Rueckert D, Sonoda LI, Hayes C, Hill DL, Leach MO, Hawkes DJ. Nonrigid registration using free-form deformations: application to breast MR images. IEEE Trans Med Imaging 1999; 18(8): 712-21.

Salat DH, Tuch DS, van der Kouwe AJ, Greve DN, Pappu V, Lee SY, *et al.* White matter pathology isolates the hippocampal formation in Alzheimer's disease. Neurobiology of aging 2010; 31(2): 244-56.

Segonne F, Dale AM, Busa E, Glessner M, Salat D, Hahn HK, *et al.* A hybrid approach to the skull stripping problem in MRI. NeuroImage 2004; 22(3): 1060-75.

Zhang Y, Wu IW, Tosun D, Foster E, Schuff N, Parkinson's Progression Markers I. Progression of Regional Microstructural Degeneration in Parkinson's Disease: A Multicenter Diffusion Tensor Imaging Study. PloS one 2016; 11(10): e0165540.
